# Combination with Annual Deworming Treatments Does Not Enhance the Effects of PCV2 Vaccination on the Development of TB in Wild Boar Populations

**DOI:** 10.3390/ani13243833

**Published:** 2023-12-13

**Authors:** Javier Galapero, Alfonso Ramos, José Manuel Benítez-Medina, Remigio Martínez, Alfredo García, Javier Hermoso de Mendoza, Rocío Holgado-Martín, David Risco, Luis Gómez

**Affiliations:** 1 Anatomy and Pathological Anatomy Area, School of Veterinary Medicine, University of Extremadura, 10003 Cáceres, Spain; jgalapero22@gmail.com (J.G.); rociohm@unex.es (R.H.-M.); luih@unex.es (L.G.); 2 Area Statistics and Operations Research Area, School of Veterinary Medicine, University of Extremadura, 10003 Cáceres, Spain; aramos@unex.es; 3 Infectious Pathology, School of Veterinary Medicine, University of Extremadura, 10003 Cáceres, Spain; jmbenimed@unex.es (J.M.B.-M.); jhermoso@unex.es (J.H.d.M.); 4 Departamento de Sanidad Animal, Grupo de Investigación en Sanidad Animal y Zoonosis (GISAZ), UIC Zoonosis y Enfermedades Emergentes ENZOEM, Universidad de Córdoba (ROR code 05yc77b46), 14014 Córdoba, Spain; remimar@unex.es; 5 Animal Production Area, CICYTEX-La Orden, 06187 Badajoz, Spain; fredgarsa@gmail.com

**Keywords:** tuberculosis, wild boar, porcine circovirus, deworming, vaccination

## Abstract

**Simple Summary:**

Tuberculosis (TB) is a highly prevalent disease in wild boar from Southwestern Spain, which in turn plays a key role in the maintenance of TB prevalence in bovine and other sympatric species. The severity of TB lesions in wild boar may be triggered by coinfections with pathogens like Porcine Circovirus type 2 (PCV2) or *Metastrongylus* spp. In fact, measures focused on the control of these pathogens (such as vaccination against PCV2 and reiterative ivermectin treatments) have proven to be effective in reducing the severity of TB in wild boar. This study aimed to assess whether a combination of deworming treatments and vaccination against PCV2 improves TB development in wild boar. The results confirmed that PCV2-vaccinated animals showed lower probabilities of suffering severe TB lesions. However, the obtained data suggest that annual deworming is not sufficient to produce a long-term parasitological load reduction that is able to influence the development of TB in wild boar, nor does it improve the effect of PCV2 vaccination on this disease. The combination of both treatments did not improve the results compared to PVC2 vaccination alone.

**Abstract:**

Vaccination against PCV2 has been proven to be an effective measure to reduce the severity of TB in wild boar. The combination of this measure with strategies focused on treating other key concomitant pathogens, such as nematodes, could be a useful strategy. This study assesses whether a combination of deworming treatments and PCV2 vaccination may reduce the prevalence and severity of TB in wild boar. The study was conducted on five game estates in mid-western Spain where four groups of wild boar were produced: control, vaccinated, dewormed and vaccinated-dewormed. Wild boars from all groups were hunted between 2017 and 2020, and all of them received a TB diagnosis based on pathological and microbiological tests. Generalised linear models were used to explore the effect of deworming and PCV2 vaccination on TB prevalence and severity. PCV2-vaccinated animals showed lower probabilities of suffering severe TB lesions. However, no differences regarding TB severity were found between dewormed and non-dewormed wild boar. PCV2 vaccination reduces TB severity in wild boar. However, annual deworming does not produce a long-term parasitological reduction that can influence the development of TB in wild boar, nor does it improve the effect of PCV2 vaccination on TB.

## 1. Introduction

Bovine tuberculosis (TB) is a chronic disease caused by bacteria belonging to the *Mycobacterium tuberculosis* (MTBC) complex, which leads to significant economic losses worldwide and poses a zoonotic risk [1]. In some European countries such as Spain, the prevalence of bovine TB has remained stable for the last decade, despite great efforts made through official eradication campaigns [2]. In this country, recent studies have linked the appearance of bovine TB breakdowns to residual infections or interactions with wildlife [3] (mainly wild boar and red deer), which display a very high prevalence of TB [4]. Thus, measures that focus on controlling TB in these wild reservoirs could be key tools in eradicating bovine TB in cattle. 

Some of the most important strategies to reduce TB prevalence in wild reservoirs include preventive actions based on biosecurity measures [5], population control through random or selective culling [5,6], and oral vaccination with heat-inactivated *Mycobacterium bovis* and *M. bovis* bacillus Calmette–Guérin (BCG) [7,8]. Furthermore, novel measures applied in wild boar to fight against TB are related to the control of concomitant pathogens like porcine circovirus type 2 (PCV2), which may decrease the severity of lesions and shedding rates in MTBC infected animals [9]. In fact, it has been shown that single dose PCV2 vaccination in wild boar reduces the extension of TB lesions in infected animals [10]. 

However, PCV2 is not the only concomitant pathogen that may influence the development of TB in this species. Wild boars coinfected with *M. bovis* and *Metastrongylus* spp. have also shown higher probabilities of suffering from severe TB lesions [11]. The existence of nematode and MTBC coinfections in wild boars may impair their ability to control TB lesion development [12]. MTBC immunological control is strongly based on a response mediated by Type 1 T-cells (Th1) and macrophages. However, the existence of nematode infections tends to generate an acquired immune response, mainly mediated by type 2 T-Cells (Th2), downregulating Th1 response [13]. Furthermore, helminths infections stimulate the production of alternatively activated macrophages with poor phagocytic activity [14]. Consequently, a recent study has shown that the application of long-term deworming programs in wild boar may also reduce the severity of TB lesions in this species [13]. The aim of this study was to explore whether the combination of deworming treatment and PCV2 vaccination in wild boar may reduce the severity and prevalence of TB in this species.

## 2. Materials and Methods

### 2.1. Study Area

The study was conducted on five game estates in southwestern Spain. Table 1 provides information about the location, area, wild boar abundance, sympatric populations, and TB status of these estates (A–E). Briefly, the studied estates range from 510 to 2100 hectares (Ha) and are fenced to prevent the dispersion of the wild boar living there. These animals are continuously fed with a specific fodder (Jabalí Mantenimiento, Iniciativas Alimentarias S.A., Ciudad Real, Spain), which is supplied ad libitum throughout the year in feeders enclosed by a fence with gates that can be activated as a selective feeding trap.

### 2.2. Experimental Design

To carry out this experiment, four groups of animals were produced: control group (C), PCV2-vaccinated group (V), dewormed group (D) and PCV2-vaccinated and dewormed group (VD). To do so, three out of five estates (A, D and E) were randomly selected to be dewormed yearly throughout the three years of experiment (2017–2019). Deworming treatments were administered using an ivermectin premix (Vectimax 6 mg/g Premix, Ecuphar, York, UK) mixed with the feeder at a concentration of 2 kg/t and supplied ad libitum during 7 days in all the fodders on the dewormed estates. To administer the PCV2 vaccination, capturing events were conducted yearly on all the studied estates. To achieve the capture, we used selective-feeding traps that allowed the entrance of wild boar, ranging between approximately four and eight months of age. Both the deworming treatments and PCV2 vaccinations were scheduled in August, when the natural feed available on the estate is scarcest, ensuring that most of the wild boar population would go in search of the fodder [15].

Captured animals were individually identified using a microchip (Pet link, Datamars SA, Lugano, Switzerland) applied subcutaneously behind the right ear and were vaccinated against PCV2 with one dose of a commercial vaccine (Suvaxyn PCV2, Zoetis, Madrid, Spain). The animals were then immediately released and allowed to return to the population. On both dewormed and non-dewormed estates, the electronically identified and vaccinated captured animals formed the vaccinated groups (V and VD), whereas animals on these estates of a similar age that had not been captured formed the unvaccinated groups (C and D). 

### 2.3. Sampling Procedure

The animals included in this study were sampled during hunting events that were conducted on all of the studied estates between October 2017 and February 2020. The age of the hunted wild boar was assessed according to teeth replacement patterns [16], and categorised as 1 (young, age <1 year), 2 (subadults, age >1 year and <2 years) and 3 (adults, age >2 years and <3 years). Hunted animals included in this study were selected according to their ages and considering the year of the experiment, such that only wild boar that could have been vaccinated (vaccinated and control groups) were sampled. Thus, during the first hunting season (2017–18), only animals of category 1 were included, in the second season (2018–19), animals of categories 1 and 2 were studied, and in the last season (2019–20), animals of all age categories were sampled. 

The existence of a microchip was checked (behind the right ear) in all of the selected animals using an appropriate reader in order to categorise them into the correct group (C, V, D, or DV). Lungs, cervical lymph nodes (submandibular and/or retropharyngeal), inguinal lymph nodes, and digestive tracts were sampled from these wild boars (when feasible) and stored at 4 °C in the laboratory for diagnostic purposes. 

### 2.4. TB Diagnosis

TB diagnosis was based on microbiological assessment from the head lymph nodes and lungs, as well as a systematic pathological examination of carcasses (following a similar methodology to that described in previous studies [10]). These TB diagnosis procedures allowed differentiation between animals with a ‘localised TB pattern’ if MTBC was confirmed in only one organ or lymph node, and animals with a ‘generalised TB pattern’ if MTBC was confirmed in more than one organ or lymph node. Furthermore, we used a pathological score based on the extent of TB lesions, ranging between 0 (no lesions) and 5 (extensive lesions), to assess the severity of TB in the studied wild boar [17].

In addition, when head lymph nodes were sectioned for microbiological culture, one of the portions was fixed in neutral-buffered formalin (4% formaldehyde) for histopathological characterisation. Haematoxylin and eosin (H&E)-stained sections were used to identify and count the TB granulomas in all their developmental stages. Four stages of granulomas (stage I, initial; stage II, solid; stage III, minimal necrosis; and stage IV, necrosis and mineralisation) were identified and labelled as previously described in cattle [18] and wild boar [19]. The degree of developmental status of the lesions was estimated for each positive animal as the main stage of all the recorded granulomas in the same lymph node.

### 2.5. PCV2 Assessment 

To confirm the effect of PCV2 vaccination on the prevalence of viral infection and viral load, DNA from the inguinal lymph nodes was extracted using a commercial kit (E.Z.N.A.^®^ Tissue DNA Extraction Systems, Omega Bio-tek^®,^ Norcross, GA, USA) following the manufacturer’s protocol. A specific and quantitative real-time PCR (RT-qPCR) assay was then conducted using a previously described protocol [20]. Duplicate reactions were run (Applied Biosystem 7300^®^, Thermo Fisher Scientific Inc., Waltham, MA, USA) for template samples (500 ng of DNA extracted from inguinal lymph nodes), standards, and non-template controls. Results were recorded as numbers of PCV2 genome copies per 500 ng of DNA. Finally, a TaqMan^®^ base real-time PCR was conducted to detect the β-actin gene using previously described protocols [21] in order to discard the presence of PCR inhibitors in the samples included in this study,.

Furthermore, a portion of the randomly selected inguinal lymph nodes was fixed in formalin 4% and subsequently cut for PCV2 immunohistochemical examination. The Avidin–Biotin Complex (Vector Elite Kit, Vector laboratories^®^, Burlingame, CA, USA) was used for immunolabelling. All samples were dewaxed in an oven (for rehydration), then treated in hydrogen peroxide 3% in methanol for 15 min to quench the peroxidase activity. Samples were washed with tris-buffered saline (TBS) 0.01M and pH 7.2. Sections were then subjected to proteolytic enzyme digestion with protease XIV (Sigma^®^, Poole, UK) at a concentration of 0.5 mg/mL for 30 min at 37 °C, following a previously described protocol [22].

Polyclonal rabbit anti-PCV2 antiserum (Porcine Circovirus Type 2 Capsid Antibody PA5-34969^®^, Thermofisher) was used at a dilution of 1:500 following the manufacturer’s recommendations. Sections were washed in TBS and then incubated for 30 min with the appropriate biotinylated secondary link antibody (Vector Laboratories^®^, Burlingame, CA, USA), which had been previously washed twice in TBS; then, sections were incubated for 30 min at room temperature with Avidin–Biotin Complex (Vector Elite Kit, Vector Laboratories^®^, Burlingame, CA, USA). Subsequently, 3,3′-diaminobenzidine tetrahydrochloride (DAB, Sigma–Aldrich^®^, Gillingham, Dorset, UK) was used for detection, and Mayer’s haematoxylin (Surgipath^®^, Peterborough, UK) was used to counterstain for 5 min. 

The results were grouped according to the number of stained cells: non-stained cells (0), low number of stained cells (1), moderate number of stained cells (2), and high number of stained cells (3) [23].

### 2.6. Parasitological Assessment

The existence of pulmonary nematodes was assessed via lung dissection. During pathological examination, the trachea and main bronchi were resected longitudinally with scissors, carefully examined, and then placed under running water to collect adult worms in a sieve. In addition, the pulmonary parenchyma was carefully dissected under a dissecting microscope to extract adult nematodes. 

In addition, stomachs and intestinal tracts were completely opened and their content was exhaustively examined to find, count, and collect all detected parasites, which were then identified following standard procedures [24].

### 2.7. Statistical Analysis

#### 2.7.1. Effect of PCV2 Vaccination and Deworming on *M. bovis* Infection and TB Severity

To explore the effect of PCV2 vaccination and deworming on the development of TB in wild boar, two sets of independent generalised linear models (GLMs) with binomial errors and logit link function were fitted, in which MTBC infection (infected or non-infected) and TB severity (localised or generalised pattern) were explained by the single, additive, and interactive effects of vaccination against PCV2, deworming, and age. Another set of GLMs with similar explanatory variables (but using a Gaussian error distribution) was checked to explain the TB pathological score (1 to 5) and the microscopic developmental stage of TB lesions. 

Of all of the proposed models, the most parsimonious one was selected to explain each response variable following an information–theoretic approach based on the Akaike Information Criterion (AIC) [25]. Statistical analysis was carried out using statistical software R version 4.0.2 (package ‘stats’). 

#### 2.7.2. Assessing the Effect of Vaccination on PCV2 Viral Load

The proportion of animals positive for PCV2 and the DNA viral load were compared between vaccinated and unvaccinated animals using the chi-square test and the Mann–Whitney U test, respectively. The level of immunohistochemistry positivity was also compared between vaccinated and unvaccinated animals using the Mann–Whitney U test.

#### 2.7.3. Effect of Deworming on Parasitic Load

The chi-square test was used to compare the frequency of the presence of lung nematodes and digestive parasites between dewormed and non-dewormed animals at the time of sampling. 

## 3. Results

### 3.1. Captures and Hunting Events

A total of 430 animals were captured, identified, vaccinated, and released in the three capture events conducted on the studied estates during this experiment (2017–2019). Forty-nine of these vaccinated animals were hunted–harvested during October of 2017 and February of 2020, constituting the V (*n* = 12) and VD (*n* = 37) groups of animals. Furthermore, another 103 animals of similar ages (but without transponders) were also hunted, constituting groups C (44) and D (59). 

### 3.2. Diagnosis and Severity of TB

Tests isolated the MTBC in 70 out of the 146 (47.94%) animals for which TB diagnosis was possible (at the time of sample, there was an absence of head lymph nodes or lung in six wild boars). Among the TB-positive animals, the mean TB pathological score was 1.79 (range between 1 and 5). Regarding TB patterns, 21 (30%) showed generalised infection, whereas 49 showed localised patterns (70%). Table 2 summarises the number of MTBC-infected animals, the percentage of TB-generalised patterns, the mean TB pathological score, and the histological development of TB lesions found in each group.

### 3.3. PCV2 Diagnosis

Testing detected PCV2 DNA in 58.49% (*n* = 77) of the animals checked (the 106 wild boars whose inguinal lymph nodes were possible to obtain) with a mean viral load of 2.3 × 10^5^ copies of PCV2 genome/500 ng DNA. Among the vaccinated groups (V + VD), the percentage of positive animals was 53.48% (20/43) with a mean load of 13,031 copies of PCV2 genome/500 ng DNA. Conversely, in the unvaccinated groups (C + D), the percentage of positive animals was 93.65% (59/63), with a mean viral load of 422,432 copies of PCV2 genome/500 ng. The frequency of animals positive for PCV2 PCR (χ^2^ = 18.662, *p*-value = 1.56 × 10^−5^) and PCV2 DNA load (W = 2279, *p*-value = 2.23 × 10^−9^) was significantly higher in unvaccinated animals (C + D). Furthermore, the level of positivity to immunohistochemistry was also significantly higher in the unvaccinated groups (C + D) (2.44) than in the vaccinated groups (V + VD) (1.44) (W = 77, *p*-value = 0.006234).

### 3.4. Parasitological Assessment

*Metastrongylus* spp. was detected in lungs belonging to 79 of the 149 animals studied (53.02%). No statistically significant differences were observed between animals belonging to dewormed estates and non-dewormed ones (52.69% vs. 53.57%). In stomachs and intestinal tracts, nematodes mainly identified as *Macracanthorhynchus* spp. and *Trichuris* spp. were detected in 126 of the 139 animals examined, with no statistically significant differences found between the dewormed and non-dewormed groups (92.13% vs. 88 %).

### 3.5. Effect of PCV2 Vaccination and Deworming on TB Parameters

**Figure 1:**
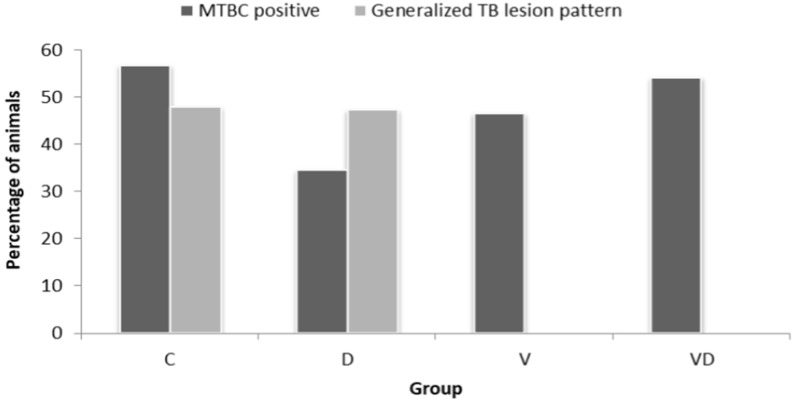
Percentage of animals infected by MTBC and suffering generalised TB pattern in different groups of animals: C (Control), D (Dewormed), V (PCV2 Vaccinated), VD (PCV2 Vaccinated and dewormed). No animals displaying generalised TB lesions were recorded in vaccinated groups (V and VD).

According to our model selection procedure (see Table 3), the most parsimonious model to explain MTBC infection in the wild boar studied was that which included age as the only explanatory variable (deviance explained = 7.1%, β = −1.46, *p*-value = 0.0002, AIC = 191.19), with a negative association between the probabilities of MTBC infection and age. Conversely, the most parsimonious options to explain both TB pattern (deviance explained 28.789%,) and TB pathological score (deviance explained = 19.3%, β = −1.2, *p* value = 0.0001) were those including PCV2 vaccination as the unique explanatory variable, showing that the existence of generalised TB patterns and TB pathological score was milder in vaccinated animals (Figure 1). The option including age as the explanatory variable was the most parsimonious model to explain the developmental stage of microscopic TB lesions (deviance explained = 4.39%, βdw = 0.02, *p*-value = 0.207).

## 4. Discussion

The results obtained in this experiment confirm that administration of single dose PCV2 vaccination in wild boar reduces the probabilities of suffering severe TB lesions in this species. However, the data obtained do not support the influence of deworming in the development of TB in wild boar.

Regarding PCV2 vaccination, our results concur with a similar experiment previously conducted in wild boar [10] that supported a negative association between PCV2 vaccination and TB severity parameters (TB pattern and TB score). The current work provides further evidence for this relationship, registering lower levels of the TB severity score (1.24 vs. 0.87) and not detecting animals with a generalised TB pattern among the vaccinated animals (Figure 1). Furthermore, our work detects a significant association between a single dose vaccination against PCV2 and a lower viral load in treated wild boar, a result also observed in the previous experiment [10]. Both the lower percentage of PCV2-positive animals and viral DNA load in the vaccinated group were corroborated by immunohistochemistry, with a lower number of positive cells in lymph nodes obtained from vaccinated wild boars.

Conversely, deworming did not show any relationship with the TB status parameters registered in wild boar populations. Several reasons could explain this finding: Firstly, the previous association between deworming and the reduction in the severity of TB lesions in wild boar was linked only with long-term treatments (longer than three years of treatment [13]), which is the time during which ivermectin was administered for this experiment. Secondly, any difference in parasitological status was observed between dewormed and non-dewormed wild boars, with high percentages of animals showing pulmonary (*Metastrongylus* spp.) and digestive (*Macracanthorhynchus* spp. and *Trichuris* spp.) parasites in both groups. This suggests that annual treatment with ivermectin for seven days at 2 kg/t is not sufficient to obtain a long-lasting improvement in the parasitological status of wild boar in these kinds of populations. The administration of ivermectin premix in a similar proportion (2.4 kg/t) produced a reduction in the parasite burden in wild boar at 14 days after treatment [26]. However, ivermectin has a limited temporary effect on treated animals, and reinfestation may occur a few weeks after application; hence, repeated treatments might be necessary to produce a longer reduction in the parasite burden in wild boar populations. In addition, ivermectin treatments have not shown a positive effect against some nematode species like *Trichuris* spp. [24]; thus, their use in combination with other drugs may be necessary to achieve satisfactory deworming. Finally, although *Metastrongylus* spp. has been associated with more severe forms of TB in wild boar, the weight of this pathogen in the statistical model was lower than the influence of PCV2 [11]. Thus, it might have been expected that the beneficial effects of deworming on the development of TB in boar would be less than what was observed after PCV2 vaccination.

Neither of the measures applied in this experiment (vaccination and deworming) were related either to the probabilities of MTBC infection or to the microscopic developmental stage of microscopic TB lesions. The histopathological development of TB lesions has been linked to the presence of concomitant nematodes in experimental studies with standardised MTBC infections [27]. However, in our experiment, the moment when MTBC infection occurs (which is a key parameter for evaluating the development of TB lesions) is unknown and randomly distributed among groups, which hinders the interpretation of results. Regarding the probabilities of MTBC infection, our findings confirm that annual PCV2 vaccination events over three years are not sufficient to decrease the prevalence of TB on wild boar estates. The long environmental persistence of MTBC, mainly in aggregation points (such as pools) [28,29] and the multi-host scenario with the existence of sympatric species infected by MTBC [30,31], could complicate the decrease in TB prevalence in wild boar [28], despite a reduction in the severity of TB and shedding rates. A longer period of application, higher vaccination efforts (with more capture events and a higher density of traps), and/or complementary control measures like TB vaccination [7], supplementation with vitamin D [32], or immunomodulant bacteria-derived products [33] and biosecurity measures may be necessary to observe an effect of PCV2 vaccination on the prevalence of TB.

## 5. Conclusions

This paper confirms the utility of PCV2 vaccination to reduce the severity of TB in wild boar populations. Although further research is necessary to understand the immunological mechanism that produces this association between PCV2 and TB, confirmation of this link highlights the importance of studying similar measures focused on controlling TB in other animal species such as cattle, or even among humans. A single annual ivermectin treatment does not seem to be an adequate complementary measure to PCV2 vaccination in order to reduce TB severity in wild boar. However, other deworming strategies, like repeated treatments or combinations of drugs, should be tested before excluding the utility of anthelmintic treatments for the purpose of controlling TB in wild boar.

## Figures and Tables

**Table 1:** Information about the estates included in this study (A-E): location (expressed as decimal degree coordinates), area (Ha), wild boar abundance (wild boar/km^2^), existence of other ungulates sympatric populations and TB status of wild boar populations (based on the percentage of macroscopic TB lesions detected in wild boar hunted during hunting season 2016–2017).

	Location	Area (Ha)	Wild Boar Abundance	% of TB Lesions in Wild Boar	Ungulate Sympatric Species
Estate A	40.009, −5.164	1021	49	30.43%	Red deer
Estate B	40.083, −5.232	2100	37	40%	Red deer
Estate C	40.125, −5.194	510	15	40%	Red deer, Roe deer
Estate D	39.632, −7.178	1600	35	55%	Red deer, Fallow deer
Estate E	39.461, −5.619	800	50	70%	Red deer, Roe deer

**Table 2:** Results of TB diagnosis, TB lesion pattern, TB pathological score, and mean stage of TB granulomas.

	MTBC Infection		TB Lesion Pattern		Mean TB Pathological Score	Mean Microscopic Stage
	Negative	Positive	Localised	Generalised		
Group C	19	25	13	12	2.45	2.66
Group D	36	19	10	9	1.95	2.78
Group V	8	7	7	0	1	3.33
Group VD	16	19	19	0	1	2.21

**Table 3:** Selection of models to explain the influence of deworming and PCV2 vaccination on MTBC infection and TB lesions development. Df: degrees of freedom, AIC: Akaike Information Criterion, ΔAIC: difference of AIC with best model, Mo: null model.

Biological Models	df	AIC	ΔAICc
Response variable: MTBC infection			
Age	2	191.1941	
Age + PCV vaccination	3	191.6725	0.4784
Age + Deworming	3	192.8113	1.6172
Age + PCV vaccination + Deworming	4	192.8737	1.6796
Age + PCV vaccination * Deworming	5	194.3952	3.2011
PCV vaccination + Deworming	3	202.5208	11.3267
Deworming	2	203.0656	11.8715
Mo	1	204.1523	12.9582
PCV vaccination	2	204.6413	13.4472
Response variable: TB pattern			
PCV vaccination	2	64.90601	
Age + PCV vaccination	3	66.89164	1.98563
PCV vaccination + Deworming	3	66.90429	1.99828
Age + PCV vaccination + Deworming	4	68.88932	3.98331
Age + PCV vaccination * Deworming	5	70.88932	5.98331
Mo	1	87.521	22.61499
Deworming	2	87.94339	23.03738
Age	2	89.47408	24.56807
Age + Deworming	3	89.92983	25.02382
Response variable: TB pathological score			
PCV vaccination	2	229.0275	
Age + PCV vaccination	3	229.9292	0.9017
PCV vaccination + Deworming	3	230.8695	1.842
Age + PCV vaccination + Deworming	4	231.7108	2.6833
Age + PCV vaccination * Deworming	5	233.645	4.6175
Deworming	2	242.0272	12.9997
Mo	1	242.0364	13.0089
Age + Deworming	3	243.4001	14.3726
Age	2	243.5965	14.569
TB lesions microscopic developmental			
Age	3	101.9311	
Age + Deworming	4	103.4529	1.5218
Age + PCV vaccination * Deworming	6	103.7277	1.7966
Age + PCV vaccination	4	103.8936	1.9625
Age + PCV vaccination + Deworming	5	105.4225	3.4914
Mo	2	169.0423	67.1112
Deworming	3	169.8573	67.9262
PCV vaccination	3	170.5336	68.6025
PCV vaccination + Deworming	4	171.5226	69.5915

## Data Availability

The data presented in this study are available in Table S1.

## Supplementary Materials

The following supporting information can be downloaded at: https://www.mdpi.com/article/10.3390/ani13243833/s1.
